# 不同中性粒细胞水平恶性血液病患者血流感染病原菌分布的特点分析

**DOI:** 10.3760/cma.j.issn.0253-2727.2023.10.012

**Published:** 2023-10

**Authors:** 晨光 刘, 爱军 廖

**Affiliations:** 中国医科大学附属盛京医院第一血液内科，沈阳 110004 Department of Hematology, Shengjing Hospital of China Medical University, Shenyang 110004, China

血流感染是一种全身感染性疾病，主要由各种病原微生物或其产生的毒素进入血液引起[1]。常见的临床表现包括发热、寒战等症状，严重的血流感染可导致感染性休克和弥散性血管内凝血（DIC）等并发症。中心静脉导管的使用以及中性粒细胞减少等因素易增加血流感染风险[2]。血流感染不仅延误患者的治疗，而且是恶性血液病患者常见的死亡原因之一，死亡率为12.9％～25.0％[2]–[5]。了解血流感染病原菌的分布情况和耐药性，对于指导早期合理使用抗生素具有重要意义。本文回顾性分析我院血液病患者的临床资料，包括血流感染病原菌的分布情况及耐药性，为早期合理使用抗生素提供参考依据。

## 病例与方法

1. 病例资料：本研究为回顾性研究，纳入2014年1月至2020年12月中国医科大学附属盛京医院血液科收治的303例恶性血液病患者。收集患者的基本情况、病原菌分布特征及其药敏结果等资料。血液病诊断标准参照《血液病诊断及疗效标准》[6]。血流感染诊断标准参照2001年《医院感染诊断标准》[7]。同一患者同一次住院多次血培养阳性病原菌视为同一病原菌，选取第1次血培养结果为分析对象。

2. 统计学处理：采用SPSS 26.0软件进行统计学分析。连续变量以中位数（*P*25，*P*75）表示，计数资料以例数（构成比）表示，不同中性粒细胞绝对计数（ANC）水平分组的多组间比较采用Kruskal-Wallis *H*检验。*P*<0.05为差异有统计学意义。

## 结果

一、患者基本情况

303例恶性血液病患者中，中位年龄 54（39，62）岁，急性髓系白血病（AML）126例（41.58％），急性淋巴细胞白血病（ALL）67例（22.11％），非霍奇金淋巴瘤（NHL）33例（10.89％），骨髓增生异常综合征（MDS）31例（10.23％），多发性骨髓瘤（MM）26例（8.58％），急性混合细胞白血病5例（1.65％），慢性髓性白血病3例（0.99％），霍奇金淋巴瘤2例（0.66％），其他11例（3.63％，包括浆细胞白血病、慢性粒-单核细胞白血病等）。共发生335例次血流感染，中位WBC为0.4（0.1，1.5）×10^9^/L，中位ANC为0.01（0，0.20）×10^9^/L。

二、病原菌总体分布情况

通过血培养共检出病原菌362株，分别为革兰阴性菌227株（62.70％）、革兰阳性菌102株（28.17％）和真菌33株（9.11％），分别有87.22％、53.92％、87.87％的菌株检出于恶性血液病患者中性粒细胞缺乏（粒缺）期间（表1）。

**表1:** 血培养阳性病原菌的分布情况

菌种	菌数（株）	构成比（%）	检出于中性粒细胞缺乏期占比（%）
革兰阴性菌	227	62.70	87.22
大肠埃希菌	73	20.16	79.45
肺炎克雷伯菌	57	15.74	98.24
铜绿假单胞菌	48	13.25	85.41
阴沟肠杆菌	16	4.41	81.25
鲍曼不动杆菌	5	1.38	80.00
其他	28	7.73	92.85
革兰阳性菌	102	28.17	53.92
凝固酶阴性葡萄球菌	53	14.64	45.28
金黄色葡萄球菌	15	4.14	26.66
屎肠球菌	12	3.31	100
其他	22	6.07	68.18
真菌	33	9.11	87.87
热带念珠菌	29	8.01	89.65
其他	4	1.10	75.00

三、不同ANC水平病原菌分布特点

根据ANC水平，将患者分为<0.1×10^9^/L、（0.1～<0.5）×10^9^/L及≥0.5×10^9^/L 3组，3组患者中分别检出病原菌243、39、80株。ANC<0.1×10^9^/L的患者主要病原菌为大肠埃希菌53株（21.81％），肺炎克雷伯菌51株（20.98％），铜绿假单胞菌32株（13.16％），热带念珠菌26株（10.69％），阴沟肠杆菌12株（4.93％），屎肠球菌12株（4.93％）；ANC（0.1～<0.5）×10^9^/L的患者检出的主要菌株分别为凝固酶阴性葡萄球菌14株（35.89％），铜绿假单胞菌9株（23.07％），肺炎克雷伯菌5 株（12.82％），大肠埃希菌5株（12.82％）；ANC≥0.5×10^9^/L的患者检出的主要菌株分别为凝固酶阴性葡萄球菌29株（36.25％），大肠埃希菌15株（18.75％），金黄色葡萄球菌11株（13.75％），铜绿假单胞菌7株（8.75％）。不同ANC水平患者检出的病原菌分布见表2，总体差异有统计学意义（*H*＝22.315，*P*<0.001），随着ANC水平升高，革兰阴性菌构成比下降，革兰阳性菌构成比增高。

**表2:** 不同中性粒细胞绝对计数（ANC）水平患者病原菌构成差异性分析

ANC水平分组	总株数	阳性菌数[株数（%）]		
		革兰阴性菌	革兰阳性菌	真菌
<0.1×10^9^/L	243	175（72.02）	40（16.46）	28（11.52）
(0.1~<0.5)×10^9^/L	39	23（58.97）	15（38.46）	1（2.57）
≥0.5×10^9^/L	80	29（36.25）	47（58.75）	4（5.00）

四、细菌耐药情况分析

1. 革兰阴性菌的耐药情况：大肠埃希菌（73株）对氨苄西林耐药率最高，为91.78％，对阿米卡星、美罗培南及亚胺培南的耐药率均为5.47％；其中产超广谱β-内酰胺酶（ESBL）的菌株42株（57.53％），耐碳青霉烯类菌株4株（5.47％）；肺炎克雷伯菌（57株）同样对氨苄西林耐药率最高，为94.73％，对阿米卡星、美罗培南及亚胺培南耐药率分别为5.26％、8.77％及8.77％；其中产ESBL的菌株18株（31.57％），耐碳青霉烯类菌株5株（8.77％）。铜绿假单胞菌（48株）对亚胺培南的耐药率最高，为16.66％，未发现对阿米卡星耐药的菌株；耐碳青霉烯类菌株8株（16.66％）（表3）。在5株鲍曼不动杆菌中检出3株耐碳青霉烯类菌株。

**表3:** 常见革兰阴性菌的耐药率（％）

抗菌药物	大肠埃希菌（73株）	肺炎克雷伯菌（57株）	铜绿假单胞菌（48株）
庆大霉素	58.90	26.31	4.16
四环素	73.97	38.59	–
氯霉素	41.09	28.07	–
复方新诺明	75.34	33.33	–
阿米卡星	5.47	5.26	0
左氧氟沙星	69.86	17.54	6.25
环丙沙星	69.86	24.56	6.25
氨曲南	42.46	28.07	14.58
氨苄西林/舒巴坦	35.61	35.08	–
氨苄西林	91.78	94.73	–
头孢吡肟	57.53	29.82	6.25
头孢他啶	32.87	24.56	4.16
头孢噻肟	68.49	33.33	–
哌拉西林/他唑巴坦	10.95	14.03	2.08
美罗培南	5.47	8.77	6.25
亚胺培南	5.47	8.77	16.66
阿莫西林/克拉维酸	12.32	15.78	–

注 –：该细菌未对该药物进行检测或只有部分菌株进行检测

2. 革兰阳性菌的耐药情况：凝固酶阴性葡萄球菌（53株）对红霉素和青霉素G耐药率最高，为86.79％，未发现对利奈唑胺及万古霉素耐药菌株，耐甲氧西林的凝固酶阴性葡萄球菌39株（73.58％）。金黄色葡萄球菌（15株）对青霉素G耐药性最高，为100％，未发现对利奈唑胺、万古霉素、环丙沙星、苯唑西林及利福平耐药菌株，未检测出耐甲氧西林金黄色葡萄球菌。屎肠球菌（12株）对氨苄西林耐药率最高，为91.66％，对庆大霉素耐药率为83.33％，未发现对利奈唑胺及万古霉素耐药菌株（表4）。

**表4:** 常见革兰阳性菌的耐药率（％）

抗菌药物	凝固酶阴性葡萄球菌（53株）	金黄色葡萄球菌（15株）	屎肠球菌（12株）
四环素	32.07	6.66	–
庆大霉素	30.18	13.33	83.33
利奈唑胺	0	0	0
复方新诺明	64.15	20.00	–
万古霉素	0	0	0
克林霉素	32.07	40.00	–
红霉素	86.79	66.66	–
环丙沙星	47.16	0	–
青霉素G	86.79	100	–
苯唑西林	73.58	0	–
利福平	7.54	0	–
氨苄西林	–	–	91.66

注 –：该细菌未对该药物进行检测或只有部分菌株进行检测

3. 念珠菌的耐药情况：共培育出33株念珠菌，其中白色念珠菌2株，光滑念珠菌1株，近平滑念珠菌1株，热带念珠菌29株。热带念珠菌对氟康唑和伏利康唑的耐药率最高，均为24.13％，对伊曲康唑耐药率为17.24％，对两性霉素B的耐药率最低，为6.89％。

## 讨论

血流感染严重威胁恶性血液病患者的生命安全。一旦发生血流感染，及早经验性应用合适抗生素，早期控制感染至关重要。本研究显示，血液科患者血流感染仍以革兰阴性菌为主，与国内外的大部分报道一致[8]–[10]，而在部分发达国家的相关报道中，血流感染则以革兰阳性菌为主[11]–[14]。虽然中性粒细胞减少是恶性血液病患者常见并发症，但是否不同ANC水平会导致致病菌的分布不同目前鲜有报道。我们的研究显示：ANC<0.5×10^9^/L的患者以革兰阴性菌感染为主，但随着ANC水平的增加，革兰阴性菌占比不断减少，革兰阳性菌占比不断增加。有研究表明[15]–[16]，血流感染病原菌为革兰阴性菌的患者初始接受不恰当抗生素治疗的死亡率较对照组明显增高，差异有统计学意义。本组病例中常见革兰阴性菌对于碳青霉烯类药物、哌拉西林/他唑巴坦及阿米卡星具有较好的敏感性，可考虑为经验治疗药物，值得注意的是，ANC<0.1×10^9^/L患者两种及以上细菌感染率可高达8.55％，当经验性应用抗生素效果不佳时，应考虑患者两种及以上细菌感染的可能。本研究中ANC≥0.5×10^9^/L患者，革兰阳性菌占比达到58.75％，治疗过程中，对于非粒缺恶性血液病患者应首先考虑革兰阳性菌感染可能性，经验性治疗则可考虑选择万古霉素及利奈唑胺。当粒缺伴发热患者出现皮肤及软组织感染、低血压等血流动力学不稳定情况、肺炎、疑似中心静脉导管相关感染等复杂临床表现时，应考虑厌氧菌、多重耐药革兰阴性菌、革兰阳性菌以及真菌感染的可能，抗生素的选择应覆盖相关可疑致病菌[17]。

近些年来，随着抗菌药物的广泛使用，细菌耐药问题日趋严重。耐碳青霉烯类肠杆菌（CRE）主要指对碳青霉烯类耐药的肠杆菌科菌种，对厄他培南、美罗培南、亚胺培南等任一碳青霉烯类药物耐药即认为CRE[18]。CRE在全球检出率呈快速增长趋势[19]。我院近7年血液科耐碳青霉烯类大肠埃希菌和肺炎克雷伯菌的检出率分别为5.47％、8.77％。Chen等[20]对于血液病患者报道的耐碳青霉烯类大肠埃希菌和肺炎克雷伯菌的检出率分别为4.2％、17.5％，与我院耐碳青霉烯类大肠埃希菌检出率基本一致。我院检出耐碳青霉烯类细菌包括大肠埃希菌、肺炎克雷伯菌、铜绿假单胞菌和鲍曼不动杆菌，其中产ESBL大肠埃希菌和肺炎克雷伯菌检出率分别为57.53％、31.57％，与陈少桢等[21]报道的产ESBL大肠埃希菌和肺炎克雷伯菌的检出率分别为55.8％和25.2％水平相似。

热带念珠菌为我院最常见的血培养阳性真菌。关于念珠菌血症，白色念珠菌仍是最常见的菌株，全球范围中非白色念珠菌株占比不断增加[22]–[24]。有研究报道免疫缺陷、中心静脉导管、广谱抗生素治疗等为真菌感染常见的危险因素[25]–[26]。国内徐春晖等[9]的研究结果同样表明热带念珠菌为成人血液病患者血培养最常见的真菌，研究表明热带念珠菌血症患者感染与中性粒细胞减少及皮肤破损有关[27]。本组热带念珠菌血症患者89.65％（26/29）发生于粒缺期间，同时86.20％（25/29）发生于AML及ALL患者。考虑粒缺、中心静脉置管和广谱抗生素的应用是此类患者易罹患真菌感染的原因。本组ANC<0.1×10^9^/L患者真菌感染占比高达11.52％，对于持续性粒缺伴发热且广谱抗菌药物治疗无效患者应考虑经验性抗真菌治疗，确诊真菌血症后及时根据药敏结果调整抗真菌药物。

综上所述，不同ANC水平患者病原菌分布特点存在明显不同，应根据患者不同ANC水平而考虑不同的经验性治疗药物，并根据是否有疗效及时调整。临床医师也应该定期回顾性分析各自中心血培养阳性病原菌的分布特点及耐药情况，进而更合理选择抗菌药物，以提高患者的治愈率并改善预后。同种病原菌根据不同ANC水平进一步分组后，部分组样本量小，探究不同ANC水平间同种病原菌耐药性是否有差异，需进一步扩大样本量。
